# Isolation in Scarlet Fever

**Published:** 1902-04-05

**Authors:** 


### ISOLATION IN SCARLET FEVER.

At a recent meeting of the Society of Medical Officers of Health, Dr. H. Malet, Medical Officer of Health of Wolverhampton, speaking on the worthlessness of current statistics concerning scarlet fever, urged that investigation of this disease should follow the lines adopted in regard to the epidemics of small-pox at Sheffield and Gloucester, namely, that instead of generalising from the varying prevalence of the disease in the whole country, account should be taken of the condition of each town and of each house and family involved. He said that in Wolverhampton he had for eight years noted the number of susceptible children in every house from which a case of the disease was notified, and has grouped the houses in two classes according as the first cases were removed to hospital or treated at home. Omitting all those houses in which there were no other susceptible individuals, and in which, therefore, no further spread was to be anticipated, he found that in 1,758 houses from which first cases were removed 4,064 susceptible children were left, while in 228 houses where the patients were treated at home there were 468 susceptibles. Of the former group only 4·8 per cent. subsequently contracted the disease, while of the latter 28·2 per cent. were attacked. Evidently this is a much more satisfactory method of demonstrating the utility of isolation hospitals in the management of outbreaks of scarlet fever than the one commonly adopted of comparing "isolation" towns with "non-isolation" towns over arbitrarily chosen periods of time. Dr. Malet's statistics definitely show that to obtain the full utility of an isolation hospital every effort should be made to isolate "first cases."

